# Does small-scale irrigation provide a pathway to women's empowerment? Lessons from Northern Ghana

**DOI:** 10.1016/j.jrurstud.2022.12.035

**Published:** 2023-01

**Authors:** Elizabeth Bryan, Dawit Mekonnen

**Affiliations:** a Environment and Production Technology Division, International Food Policy Research Institute, 1201 Eye Street NW, Washington, DC, 20005, USA; b Environment and Production Technology Division, International Food Policy Research Institute, IFPRI C/o ILRI P.O. Box, 5689, Addis Ababa, Ethiopia

**Keywords:** Gender, Women's empowerment, Irrigation, Impact evaluation, Ghana

## Abstract

Given persistent gender inequalities that influence how the benefits of technologies are distributed, the expansion of small-scale irrigation technologies requires the consideration of important gender dynamics and impacts. Women's lack of agency and access to resources relative to men, and other social constraints, often limit their ability to adopt and benefit from agricultural technologies. At the same time, expanding access to agricultural technology to women may provide a pathway for empowerment. This paper explores the potential for small-scale irrigation technologies to increase women's empowerment by evaluating the impacts of an intervention that distributed motor pumps to small groups of farmers in Northern Ghana. The paper draws on two rounds of survey data that included the Women's Empowerment in Agriculture Index, before and after the motor pump intervention was implemented. To control for possible selection bias at the baseline, the difference-in-difference method is used to estimate the impact of the program on indicators of women's empowerment. Spillover effects are estimated by comparing outcomes of farmers in treatment villages that did not receive the pumps with farmers in control villages, where no motor pumps were distributed. The results show no significant impact of the program on measures of women's empowerment. However, there are potential negative impacts, including among households that did not benefit from the intervention. The results highlight the need to pair interventions that distribute agricultural technologies with complementary investments in infrastructure that increase access to water for irrigation, as well as other activities and approaches that ensure women can reap the benefits.

## Highlights

• Supplying men and women farmers with motor pumps for small-scale irrigation did not increase women's empowerment.
• Rather, the intervention had negative spillover effects on women from households that did not receive pumps.
• Women's empowerment in the study communities is generally increasing over time irrespective of the irrigation intervention.
• The benefits of the motor pump intervention for women are indirect, including increasing household asset holdings.
• More careful planning and implementation of irrigation interventions are needed facilitate women's empowerment.

## 1 Introduction

Small-scale irrigation is a key strategy for increasing the agricultural output and incomes of smallholder producers in many countries in sub-Saharan Africa (Xie et al., 2014; Burney et al., 2010). Other benefits of irrigation include improvements in food security, nutrition, and health outcomes by increasing household disposable income, by diversifying production toward more nutrient rich foods, such as fruits and vegetables, and by increasing household access to a domestic water source (Mekonnen et al., 2019; Xie et al., 2018; de Fraiture and Giordano, 2014; van Koppen et al., 2014; Namara et al., 2005; Frenken, 2005). Irrigation can also increase climate resilience by reducing production risk and increasing food availability throughout the year, including during the lean season, leading to improved dietary diversity (Baye et al., 2022). While irrigated area remains small (only 4 percent of cultivated area) in sub-Saharan Africa, there is considerable potential for expansion in many parts of the region, and private investments in small-scale irrigation technologies are proliferating (Giordano et al., 2012; de Fraiture and Giordano, 2014).

Irrigation investments in Ghana have largely targeted large-to medium-scale schemes, however, more recent emphasis has been given to the development of smaller schemes and infrastructure and partnering with the private sector to expand access to technologies for small-scale irrigation (MoFA, 2018). Small-scale irrigation is defined as irrigation that farmers control themselves for cultivating small plots of land, using technologies and systems which they operate and maintain at the household level or in small groups or communities (Bryan and Lefore, 2021). Despite these policy efforts, actual diffusion of technologies for small-scale irrigation remains slow due to the high upfront cost of the technologies, lack of availability of the technologies and supporting services (e.g. financing and extension), lack of coordination among stakeholders, and the limited engagement of farmers in the diffusion process (Balana et al., 2020; Minh et al., 2020; Atuobi-Yefoah, Aberman, and Ringler, 2020).

There are important gender dynamics to be considered with the expansion of small-scale irrigation technologies. Women's lack of access to resources and more limited agency relative to men, and other social constraints, often limit their ability to adopt agricultural technologies (Deere and Doss, 2006; Doss and Morris, 2001; Peterman et al., 2014). In the case of irrigation, women face barriers to adoption, such as lack of access to agricultural land, lack of labor for irrigation, greater time burden, and social norms that prohibit women's use of particular types of irrigation technologies, such as treadle pumps (Nation, 2010; Van Koppen, Hope, and Colenbrander, 2013; Namara et al., 2014; Njuki et al., 2014; Imburgia, 2019; Lefore et al., 2019). Moreover, once adopted, the costs and benefits of agricultural technologies may not be equally distributed across different household members (Theis et al., 2018). The extent to which women benefit from new agricultural technologies, like motor pumps, depends in part on their bargaining power in determining how technologies are applied and used, who provides the labor, and who controls the agricultural output and income from the sale of irrigated crops (ibid).

While women often face greater constraints in adopting, using, and benefitting from irrigation technologies, some case studies show that when women overcome these constraints, they benefit in important ways (Bryan and Lefore, 2021). Several studies suggest that increasing women's access to small-scale irrigation for home garden production, can provide economic opportunities for women, reduce their time burden, and increase assets and income controlled by women (Graham et al., 2016; van Houweling et al., 2012; van den Bold et al., 2013; Burney et al., 2010). Conversely, irrigation has the potential to disempower women relative to men to the extent that it increases their workload or limits their input into production decisions and control over the use of income from irrigated production (Nation, 2010). Thus, understanding the circumstances under which small-scale irrigation provides an opportunity for women's empowerment deserves greater attention.

Much of the existing literature on women's empowerment explores the ways in which women's empowerment contributes to other development outcomes, such as improvement in agricultural productivity (Seymour, 2017), increase in production diversity (De Pinto et al., 2020) or improved food security and nutrition (Malapit and Quisumbing, 2015; Kassie et al., 2020). A growing body of literature examines the extent to which development interventions, including those that aim to increase women's access to and control over productive assets, support women's empowerment (Cornwall, 2016; Johnson et al., 2016; Malapit et al., 2019; Anderson et al., 2021). Conceptual framing of women's empowerment in the literature has largely converged around the definition of Kabeer (1999), which describes empowerment as a complex process of change, whereby women access resources and exercise agency leading to improvements in their well-being outcomes and other achievements. Women's empowerment is multidimensional and complex—while some aspects, such as objective outcomes and achievements, are easier to measure and observe; other aspects, such as agency, can be subjective and more difficult to measure (Laszlo et al., 2020). Nevertheless, tools to capture the multiple dimensions of women's empowerment that are linked to the theoretical concepts, including various aspects of women's agency, have been developed and are becoming more widely used (Malapit et al., 2019). These tools, including the Women's Empowerment in Agriculture Index (WEAI), have evolved over time into a set of comparable and complementary indicators to diagnose and monitor changes in women's empowerment and have been widely used across contexts, offering consistency in empowerment measurement (Alkire et al., 2013; Malapit et al., 2017, 2019; Martinez et al., 2021).

More recent applications of the WEAI focus on measuring women's empowerment as an outcome of development interventions that aim to reach, benefit, and empower women and have been applied across a range of contexts with varied results (Johnson et al., 2018; Malapit et al., 2019). Some recent examples include a study by Kumar et al. (2021) that finds that women's participation in self-help groups in India increases women's empowerment scores and gender parity, largely through an increase in their control over income, decision-making over credit, and active involvement in groups. Crookston et al. (2021) examine the impact of a set of interventions designed to increase resilience on measures of men's and women's empowerment in Burkina Faso and find that the intervention protected program participants from empowerment losses due to an economic shock during the study period. Quisumbing et al. (2021) found that an intervention that provided agricultural, nutrition, and gender-sensitization trainings to men and women in Bangladesh increased women's empowerment outcomes.

This paper examines the extent to which an intervention that provided motor pumps for small-scale irrigation to groups of men and women farmers in Northern Ghana affected measures of women's empowerment using indicators from the Abbreviated Women's Empowerment in Agriculture Index (A-WEAI) developed by Malapit et al. (2017). The analysis focuses on two composite measures of women's empowerment, an individual empowerment score and the number of adequacies achieved across several measures of empowerment, as well as indicators of key aspects of women's empowerment thought to be affected by the intervention: women's input into production decision-making, control over assets, control over income, and work burden. The study also uses qualitative data collected through focus groups and interviews with farmers in the study communities to understand the quantitative results. Results shed light on the extent to which expanding access to irrigation technology on its own benefits and empowers women and points to potential pitfalls of this approach.

## 2 Background

### 2.1 Policy context

Public irrigation investments in Ghana have been largely targeted towards large-scale irrigation schemes; however, these schemes demonstrated limited effectiveness in terms of economic benefits to farmers, due to poor construction, operation and maintenance, lack of support services, and land insecurity (Owusu, 2016; Dittoh et al., 2013; Laube et al., 2012). These large-scale schemes complement the development of hundreds of small reservoirs that have been constructed to capture rainwater for multiple uses, including irrigation (Acheampong et al., 2018) as well as more recent interest in other water sources, including groundwater and water harvesting schemes (Adam et al., 2016; MoFA and GIDA, 2012). However, current policy guidance still emphasizes increasing public investment in developing and rehabilitating irrigation schemes and infrastructure (MoFA, 2010; MoFA, 2018).

Many smallholder farming households have already developed their own rudimentary irrigation facilities and practices, typically consisting of hand-dug wells and buckets for extracting and applying water for irrigation. Few smallholder farmers use modern irrigation technologies (Dittoh et al., 2013; Laube et al., 2012). There is little direct institutional and policy support for private farmers investing in small-scale irrigation, as this is considered the domain of the private sector (Namara et al., 2010, 2014; Owusu, 2016). Moreover, there is a lack of official information on the area covered by ongoing private small-scale irrigation and on the extent of proliferation of small-scale irrigation pumps throughout the country (Namara et al., 2010, 2011, 2014; Owusu, 2016). However, studies suggest that small-scale irrigation covers 14 times more land than the area covered by public irrigation schemes and benefits 500,000 smallholder farmers in the country (Namara et al., 2014).

Private sector investment is increasing in the areas of importation, manufacturing, and retailing of irrigation equipment (Minh et al., 2020; Atuobi-Yeboah et al., 2020). However, most imported products are targeted towards large-scale commercial farms and the scale of locally-manufactured irrigation equipment targeted to smallholders remains too limited to accelerate the diffusion of irrigation technologies (ibid). Retailers that distribute irrigation equipment, such as motor and manual pumps, water tanks, and PVC pipes, are typically located in large urban areas and often depend on partners, such as NGOs, to distribute equipment to farmers (ibid). Moreover, irrigation equipment supply chains are not gender-sensitive—technologies are not designed and distributed in ways that meet the needs of women (Minh et al., 2020). Both men and women farmers lack knowledge and access to service providers to support the installation, operation, and maintenance of irrigation equipment (ibid).

Given the important role women play in the agriculture sector, the Gender and Agricultural Development Strategy (MoFA, 2015) provides a detailed framework for integrating gender into agricultural policies, programs, and projects. While the strategy does not mention irrigation specifically, it highlights the need to develop and disseminate gender-sensitive technologies along agricultural value chains (ibid). In line with these mainstreaming efforts, Ghana's Irrigation Policy acknowledges the importance of increasing women's access to land and water as well as their participation in local water management organizations. However, gender mainstreaming in policy has not yet translated into practice and new and improved technologies are much less likely to be disseminated to women (MoFA, 2007) and there is currently little coordination or oversight of private sector and development partners to ensure equitable and efficient diffusion of small-scale irrigation technologies (Minh et al., 2020). Both men and women smallholder farmers remain constrained in adopting technologies for small-scale irrigation, such as motor pumps, because of the high initial investment cost, despite the potential to increase farm profits and incomes (Balana et al., 2020; Wrigley-Asante et al., 2017).

### 2.2 Intervention design and theory of change

While efforts to expand small-scale irrigation are ongoing, access to motor pumps in many parts of the country remains extremely limited. The technologies are generally not available in local markets, including in the Upper East Region. Moreover, motor pumps are unaffordable for many smallholder farmers and farmers lack credit access to facilitate adoption of motor pumps. Thus, there are virtually no sales of motor pumps in the study area, though some farmers buy pumps from traders who cross the border from Togo. This research evaluates the impact of an irrigation intervention implemented in the Upper East Region of Ghana by International Development Enterprises (iDE), through which small groups of men and women famers were provided preferential loans to adopt water extraction technologies (motorized diesel pumps) for irrigation during the dry season. The pumps distributed to farmers cost 890 cedis (just over US$200 at the time when pumps were distributed in 2016), and farmers were expected to repay the loan in several installments following the dry season harvest. In addition to the cost of the pump, farmers paid an average of US$37 per season for fuel. Thus, the intervention took a market-based approach to expanding small-scale irrigation technologies by targeting key constraints to motor pump adoption—credit and technology access. The ultimate objectives of the intervention were to increase productivity of dry season agriculture, improve diets and food security, and contribute to women's empowerment.

While the project did not include any activities that were intended to challenge patriarchal norms, such as facilitated dialogues, the distribution of an important productive asset (the motor pump) could lead to women's empowerment through four pathways. First, the project assumed a high degree of participation in the project by women because women in the target communities are often responsible for dry season farming as men engage in other commercial activities, such as cross-border businesses. This assumption was later confirmed as the majority of farmers in the project were women. Thus, the project would empower women directly by improving their access to and control over productive assets. Second, the project assumed that motor pumps would enable women to expand production of high-value crops and increase the income they generate from dry season farming. This is because existing dry season farming in these communities typically involves using small buckets or jerricans to bring water from the source to the fields—a particularly inefficient method of irrigation that limits the extent and profitability of irrigated production. Third, the project hypothesized that having access to motor pumps would decrease women's time burden by reducing the amount of time they spend irrigating or collecting domestic water using buckets. Fourth, the project expected that more women having access to and control over modern irrigation technologies and income from irrigation would increase their decision-making authority in the household, including over important production decisions. In addition to these expected impacts on women's empowerment, the project envisioned that both women and men would also benefit from improvements in food and nutrition security, due to greater availability of irrigated produce and more income for food purchases.

The project also anticipated potential challenges to achieving gender equity in program outcomes, including women's more limited access to land and water resources and the risk that men may take over irrigation technologies and activities if they become more profitable. Other identified risks include that woman would be less likely to adopt the technology despite having won the lottery, due to higher risk aversion to accept the loans being provided for the purchase of motor pumps and the associated re-payment requirements. The project aimed to minimize this risk by offering more favorable terms than other credit providers in the area, including a lower interest rate and a delay in repayment until after the harvest.

In each village, farmers were directed by iDE to self-organize into groups to receive a group loan to purchase a motor pump. All farmers in the community were invited to participate in the intervention on a voluntary basis. Both men and women joined groups so that the final set of groups included some same-sex groups and some mixed groups. In some cases, multiple members of the same household joined groups. Overall, two-thirds of the group members were women, but the number of women participants varied across communities. The precise number of groups formed in each village depended on the amount of water available for irrigation and the level of interest among farmers.

Farmers first organized into “confidence” groups of around 20 farmers to receive group loans from a micro-finance institute to purchase agricultural inputs, such as seeds and fertilizers. A total of 42 confidence groups were formed across villages. The confidence groups were further broken down into smaller “trust” groups of 5 farmers each. A sub-sample of trust groups was randomly selected to receive a loan to purchase a motor pump through a lottery process in a subset of villages. Thus, the level of randomization was at the trust group level and the lottery was conducted during a meeting with most members of each trust group present. Therefore, not all trust groups within the larger confidence groups would receive a pump through the lottery. Given liquidity constraints, microfinance organizations are typically not able to provide loans large enough for farmers to purchase motor pumps. Therefore, iDE managed financing of the loans, which could only be used for the purchase of a motor pump to be shared by members of the group. Trust groups that won the lottery were expected to share the pump amongst all group members and each member was expected to contribute to repayment of the loan.

The intervention faced some challenges in implementation. First, pumps were distributed to trust groups that won the lottery in December of 2015, after the dry season cultivation had already started. Therefore, farmers were not able to utilize the pumps to increase irrigated area and profits during the first year of the program and were unable to repay the loans following the dry-season harvest. Several farmer groups returned the pumps to iDE for later re-distribution before the next dry season (beginning in September 2016). Second, while most groups repaid the full loan amount, some groups did not complete payments and returned the pumps to iDE. Furthermore, there were irregularities in the re-distribution of the pumps in September 2016 that resulted in some farmer groups not receiving the pumps as intended. At the end of the program approximately 60 percent of the farmer groups had received the pumps and fully repaid the loans. Moreover, while the program intended all 5 trust group members to share the pump equally, some group members reported to the research team that they had less access to the pump, although individual pump use was not tracked by the program implementors.

## 3 Data

### 3.1 Study area

The intervention was carried out in the Garu-Tempane District in the Upper East Region of Ghana. This region is characterized by one rainfall season followed by a long dry period. Irrigated cultivation largely takes place during the dry season. The most important crops produced with irrigation in this area are onions, okra, tomato, pepper, watermelon, and leafy green vegetables, all of which are considered high-value crops (Mekonnen et al., 2019). These crops are sold in local markets by women vendors, and some (especially onions) are bundled and transported (typically by male traders) to larger urban centers, namely Accra and Kumasi (IFPRI, 2020).

The survey data show that the main sources of water for irrigation include groundwater, typically extracted from the dry riverbed through hand-dug wells, and surface water bodies, such as small reservoirs, dams, and ponds. In communities where hand-dug wells are needed to obtain water (as opposed to small reservoirs or dams), men are responsible for digging the wells in the dry riverbed every season, while women assist with irrigating plots located near the water source using buckets or jerricans (Bryan and Garner, 2022). Most households used rudimentary methods to apply water to their fields, namely jerricans, buckets, and hoses. Other irrigation application methods include flooding, furrow, and level basin (gravity) and a few households used more water efficient methods, such as sprinkler or low-cost drip. At the start of the intervention, very few households in the study area used motor pumps or even manual pumps to extract water for irrigation.

Irrigators that use any type of pump or gravity irrigate more land, and hence have higher potential for increased income and productivity gains. While most farmers, including those that irrigate and those that do not, report using their own seeds, irrigating households were more likely to report purchasing local seed, including improved seeds. Farmers reported that the main constraints to dry season agriculture include plant disease, insect damage, insufficient water, and, to a lesser extent, weeds. Most farmers irrigated twice per day and spent an average of 2.5 hours per irrigation.

There are clear gender-differentiated livelihood and farming roles in the study area. Women assist their husbands with the main household plots but often cultivate their own plots of land allocated to them by their husbands or other family members (Bryan and Garner, 2022). There are some crops grown by both men and women, but women tend to select different crops on the plots that they cultivate themselves, such as leafy greens, given their consumption preferences (ibid). Women participate in irrigated production and often provide the labor for irrigation, which is particularly time consuming with manual methods.

Women also participate in alternative livelihood activities, including petty trading, shea butter and groundnut processing, and brewing and selling the local drink *Pito*, basket weaving, and other activities (Bryan and Garner, 2022; Lawson et al., 2020; Lolig et al., 2014). Women are also responsible for the sale of crops produced by the household in local markets and tend to dominate market transactions, except in the case of livestock. Because women do not own land but access it by borrowing from their husbands or other family members, this limits their ability to make decisions regarding production and affects their willingness to apply agricultural inputs and adopt new agricultural practices, especially ones that require technical knowledge and upfront investment, like small-scale irrigation (Bryan and Garner, 2022; Lawson et al., 2020).

### 3.2 Data and sampling frame

This paper uses household and intra-household survey data collected from selected sites in Ghana by IFPRI and the University of Development Studies in Tamale, Ghana. The baseline survey was carried out between early November 2015 to early February 2016 in 9 villages in Garu-Tempane District in the Upper East Region of Ghana where a total of 800 households were interviewed across these communities. The endline survey round was conducted between December 2017 and February 2018, covering 759 households. The intervention, whereby pumps were distributed to 42 trust groups of farmers through a random lottery, took place after the baseline round was completed.

Qualitative data were collected in between survey rounds, through life history interviews and sex-disaggregated focus groups with men and women farmers and traders in a selected set of treatment and control villages. The qualitative protocols were designed to complement the survey modules and intended to support quantitative analysis of the pathways of impact. Focus groups covered local understanding of empowerment, gendered livelihood roles, and experiences with the irrigation intervention, while life history interviews explored gender roles, decision-making, intra-household dynamics, relationships with the community, and perceptions of self and aspirations. For more details on the qualitative approach and analysis see Bryan and Garner (2022, 2020).

This region was selected for the intervention given its location near the iDE area of operation where similar interventions were implemented; but farmers in the selected villages were not previously reached by any irrigation intervention. The area was identified as having high potential for irrigation expansion based on an ex-ante analysis. The sampling frame was based around the iDE intervention, whereby households that self-selected into the intervention were randomly divided into treatment and control groups. Because farmers self-selected into groups, the number of participants that volunteered to participate in the intervention was uneven across villages. In some villages there were as few as 20 program participants and in the largest community (comprised of several adjacent villages) there were close to 300 participants. Given the large differences in the number of farmers and trust groups across villages, assignment of treatment and control villages was based on a random selection of paired villages with similar levels of program participation. This was done based on the assumption that differences in voluntary program participation reflected differences across communities—e.g. that villages where participation was high were likely to have better access to water resources for irrigation and more households irrigating at baseline.

After the program was introduced, farmers organized into groups and the baseline survey was completed, households were assigned to treatment and control groups based on the random selection of treatment villages^1^ and lottery-winning trust groups^2^ within treatment villages. Not all households that originally joined groups and participated in the baseline survey ended up participating in the lottery (see the last 2 columns of Table 1). Out of the 412 group participants in the intervention villages, iDE records showed that 351 people participated in the lottery, of which 171 were in trust groups that won.

**Table 1:** Distribution of farmers, trust groups, and lottery winners across villages.

Community	Assignment	# of farmers	# of trust groups	# of lottery participants	# of lottery winners
Mognoori	Treatment	295	59	250	121
Akara	Control	157	31	n/a	n/a
Gbanterago Alemgbek	Control	114	23	n/a	n/a
Asikiri	Control	58	11	n/a	n/a
Yidigu	Treatment	57	11	49	24
Denegu	Treatment	39	8	38	19
Bugri Natinga	Control	39	7	n/a	n/a
Binipiala	Treatment	21	4	14	7
Zule	Control	20	4	n/a	n/a
Total		800	158	351	171

This results in the following groups of households in our sample: 1) lottery winners in early treatment villages, 2) lottery losers in early treatment villages (and non-participants), and 3) farmers who formed groups in control villages that did not participate in the lottery. Spillover effects can be determined by comparing the outcomes of households from trust groups in treatment communities that did not win the lottery with households from groups in control communities.

The survey included household-level modules on agricultural activities, socio-economic characteristics, livelihoods, nutrition, health, and food security, among other topics. The survey also included a modified version of the Women's Empowerment in Agriculture Index (WEAI) in the baseline survey and a modified project-level WEAI (pro-WEAI) in the endline to measure the relationship between women's empowerment and irrigation. Common modules between these two versions of the WEAI comprise the Abbreviated-WEAI (A-WEAI), which is used for the analysis. The WEAI is an intra-household survey-based tool, asked of both the main male and female decisionmakers in a household used to determine inclusion of women in domains important to the agricultural sector. It was modified for this study to include more questions and response codes related to irrigation. In addition to the original WEAI modules, additional questions on credit, savings, group membership, access to information, and access to extension were asked of both men and women respondents from the same household.

## 4 Methods

**Fig. 1:**
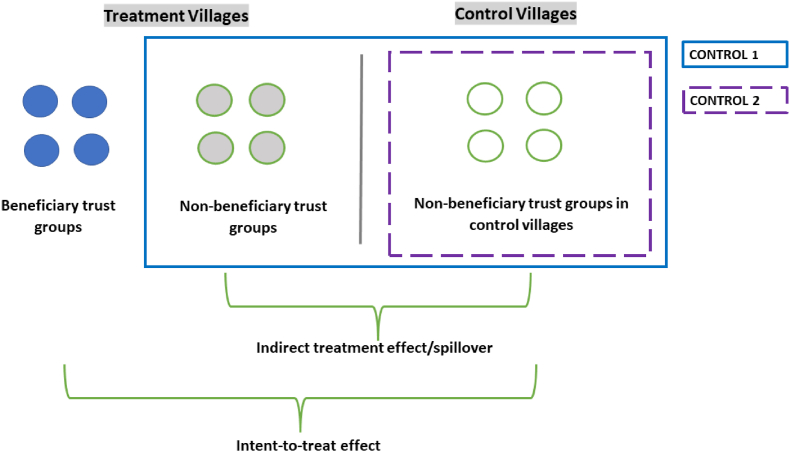
Experimental design.

The treatment group is composed of households in trust groups that won the lottery to receive a motor pump, in villages where the lottery was carried out. We estimate the intent-to-treat effect, regardless of whether the households in the lottery-winning trust groups actually used the pump, given inconsistencies in pump distribution and repayment across lottery-winning groups, and uneven sharing among trust group members. The intervention was designed to provide two alternative control groups to evaluate the impacts of the program on indicators of women's empowerment. The first control group (control 1) is comprised of those households in trust groups that did not win the lottery as well as those in villages where no lottery was held. However, if the pumps were shared outside of the winning trust groups, households within treatment villages may have gained access to motorized pumps even if they did not participate in a group that won the lottery. Given the possibility of such spillover effects within treatment villages, a second control group (control 2), comprised only of households in control villages, was also used. Fig. 1 illustrates these two different control groups: control group 1 is shown by the larger outline, while control group 2 is shown by the dotted outline.

To control for possible selection bias at the baseline, the difference-in-difference method is used to estimate the impact of the program on indicators of women's empowerment:

Y_i_ = β_0_ + β_1_T_i_ + β_2_R_i_ + β_3_T_i*_R_i_ + β_x_X_i_ + ε

Y represents the set of measures of women's empowerment. In the case of the difference-in-difference estimation, these outcomes are the individual A-WEAI score (composed of 6 sub-indicators), the number of A-WEAI sub-indicators for which the individual achieved adequacy (0–6), and the set of sub-indicators for input into production decisions, control over assets, control over income, and work balance. β_1_ accounts for the difference between the treatment and control groups prior to the intervention, β_2_ captures the trend over time (R signifies round), β_3_ captures the intent-to-treat effect. The model also includes a set of control variables (X_i_) comprised of individual and household characteristics, and village-level dummy variables. Baseline levels were used for some control variables thought to be influenced by the intervention. Individual control variables include age, whether the participant had any formal schooling, religion, and whether the household had multiple wives. Household level controls include household size, number of children under 5, size of agricultural landholdings at baseline, distance of the plot from the household, tropical livestock units (TLU) at baseline, irrigation status at baseline, source of water, and experience with shocks. Standard errors are adjusted for stratification by paired villages and clustered by trust groups (158 groups) within the larger confidence groups (42 groups). The same model is run for both sets of control households (control 1 and control 2) as described above.

Spillover effects are also estimated by comparing outcomes of members of non-beneficiary trust groups in the treatment villages with non-beneficiary groups in control villages. The spillover estimation includes a control for the intensity of the potential spillover that is defined as the number of trust groups that won the lottery within the larger confidence group.

### 4.1 Baseline characteristics

The A-WEAI is an aggregate index comprised of two subindices: the five domains of empowerment (5DE) and the gender parity index (GPI) (Malapit et al., 2017). The 5DE is based on 6 weighted sub-indicators of various aspects of women's empowerment covering 5 domains: 1) input into agricultural production decisions, 2) access to and control over productive resources, 3) control over income, 4) community leadership, and 5) time allocation (Alkire et al., 2013). The sub-indicators are adequacy scores, which take the value of one if a woman achieves adequacy in that indicator or zero if she does not. The six sub-indicators of women's empowerment within the 5 domains of empowerment are: input into productive decisions, ownership of productive assets, access to and decisions on credit, control over the use of income, group membership, and work burden. The weights given for each indicator in the construction of the 5DE are shown in Table 2.

**Table 2:** A-WEAI domains, indicators, and weights.

Domains	Sub-indicators	Weight
Production	Input in productive decisions	1/5
Resources	Ownership of assets	2/15
	Access to and decisions on credit	1/15
Income	Control over use of income	1/5
Leadership	Group membership	1/5
Time	Work balance	1/5

One of the benefits of the WEAI tools is that they are decomposable into sub-indices, sub-indicators, and population subgroups (Alkire et al., 2013; Malapit et al., 2017). A selected set of outcome indicators was used to assess the impacts of the intervention on women's empowerment based on the theory of change for the intervention (definitions are shown in appendix 2). These include aggregate measures: the A-WEAI individual empowerment score (5DE) composed of the 6 sub-indicators shown in Table 2 and the number of adequacies achieved (0–6). We also assessed the impact of the intervention on 4 key sub-indicators that were hypothesized outcomes of the intervention: input in production decisions, ownership of productive assets, control over income, and work balance.

In addition to the outcome indicators of women's empowerment, we explore whether household assets increased as a result of the irrigation intervention by including a variable for the number of household asset types owned by the household. Asset categories include large livestock, small livestock, poultry and other small animals, fish pond/fishing equipment, farm equipment (non-mechanized), farm equipment (mechanized), non-farm business equipment, house of building, large consumer durables, small consumer durables, cell phone, non-agricultural land, means or transportation, irrigation pond or tank, and irrigation pump. The last two asset categories were added to the modified WEAI to strengthen focus on irrigation.

A comparison of the average value of the dependent variables revealed no statistically significant differences at the baseline between women in households assigned to treatment and control group 1 as shown in Table 3. Weighted individual empowerment scores (5DE) for women were 0.69 in control group 1 and 0.72 in the treatment group and the average number of adequacies achieved was approximately 4 for both treatment and control groups. Women were more likely to achieve adequacy in having input into agricultural decisions and control over income at the baseline compared to asset ownership and work balance. However, balance tests using control group 2 show significant differences between treatment and control with respect to women's involvement in agricultural decision-making, whereby women in the treatment group are more likely to achieve adequacy in this indicator.

**Table 3:** Summary statistics of dependent and independent variables at baseline for women in treatment and control households.

Dependent vars	Treat (obs = 139)	Control1 (obs = 370)		Control2 (obs = 238)	
5DE score	0.72	0.69		0.68	
Number of adequacies	4.07	3.98		3.97	
Input into agricultural decisions	0.89	0.85		0.83	*
Asset ownership	0.55	0.56		0.54	
Control over income	0.91	0.89		0.86	
Work balance	0.53	0.52		0.49	
Number of asset types owned	5.49	5.85		5.84	
Independent vars					
Household size	6.96	6.8		6.93	
Children under 5	0.97	0.78	*	0.79	*
Land size (acres)	7.39	6.47	**	6.44	*
Average plot distance (mins)	25.54	22.52		19.68	**
Tropical livestock units (TLU)	3.22	3.69		4.05	
Household has irrigated plots	0.7	0.69		0.68	
Household has groundwater for irrigation	0.47	0.49		0.48	
Household has surface water for irrigation	0.23	0.21		0.21	
Experienced climate shock	0.49	0.43		0.50	
Experienced idiosyncratic shock	0.4	0.46		0.42	

***p < 0.01, **p < 0.05, *p < 0.1.

Some household-level independent variables did differ across treatment and control groups 1 and 2 at the baseline. Households in the treatment group were more likely to have more children under the age of 5 and were more likely to have larger land holdings. In the case of control group 2, these households were significantly more likely to have shorter distance to plots than the treatment group. There were no differences with respect to average household size, whether the household irrigated at baseline, livestock holdings, sources of irrigation water, and experience with shocks. The fact that there were few statistically significant differences suggests that the treatment and control groups were relatively well balanced at baseline.

There were larger differences between the spillover group and control group 2, which suggests that the randomization failed to achieve balance in these groups at baseline (results shown in Appendix Table 1.4). Women in the spillover group were more likely to have achieved adequacy in input into agricultural decisions, control over income, and work balance at the baseline compared to control group 2. We, therefore, suggest the spillover results be treated with some degree of caution.

Given that they survey did not have complete WEAI data for every household in the sample and because of attrition between rounds 1 and 2, there are fewer observations in the endline and panel datasets used for the analysis, and households assigned to the control group were more likely to have dropped out between rounds. Therefore, the results are weighted to account for possible attrition bias using inverse probability weights, based on baseline characteristics (sex and literacy of the household head, household size, and irrigation status at baseline) and treatment status (i.e. whether the household was assigned to the set of treatment villages and whether they won the lottery to receive a pump). The results with and without the weights for attrition were robust—the direction and significance of the impact estimates remain consistent.

## 5 Results

### 5.1 Impacts of the intervention on measures of women's empowerment

Different estimations were run comparing women assigned to the treatment group with both alternative control groups. Both sets of results show no significant impacts of the program on aggregate indicators of women's empowerment (A-WEAI score and number of adequacies) or any of the sub-indicators (Table 4). This suggests that while the intervention may have provided some benefits to lottery-winning households, and indirectly to women, it did not influence women's agency. That is, while women may have benefitted indirectly from an increase in profits from irrigated production and household income following the introduction of motorized pumps, women's control over income decisions, influence over production decisions, control over assets, and work burden were not affected. Descriptive and qualitative results suggest that women may also have benefitted from a reallocation of household budgets—some women reported not having to purchase vegetables in the market given greater availability of vegetables from own production (Bryan and Garner, 2022). While descriptive survey results indicated that pump users sold most of their irrigated output, most also kept a small portion for household consumption. As a result, 39 percent of households who won the lottery reported consuming different foods because of having access to the pump. Pump users also reported using the income from the sale of irrigated crops to purchase other foods (32 percent), pay school fees (66 percent), and pay medical expenses (46 percent). However, these benefits were not enough to significantly increase women's bargaining power.

**Table 4:** Difference-in-difference intent to treat effect on indicators of Women's empowerment and household assets, alternative control groups.

	A-WEAI score	No. Of adequacies	Production decisions	Ownership of assets	Income decisions	Work balance	No. Of asset types owned
	Control 1						
TreatxRound	0.00124	0.0999	−0.00467	0.145	0.0700	−0.0805	0.258
	(0.0158)	(0.100)	(0.0453)	(0.0792)	(0.0428)	(0.0473)	(0.221)
Treat	0.0507***	0.252**	0.0807***	0.0197	0.0109	0.0308	−0.439**
	(0.0125)	(0.0697)	(0.0198)	(0.0433)	(0.0489)	(0.0434)	(0.151)
Round	0.0904**	0.624***	−0.0904	0.198**	0.00880	0.270***	0.894***
	(0.0244)	(0.124)	(0.0686)	(0.0499)	(0.0268)	(0.0403)	(0.196)
Observations	770	770	770	770	770	770	770
R-squared	0.140	0.156					0.198
	Control 2						
TreatxRound	−0.0189	0.0356	−0.0445	0.0552	0.0353	−0.0858	0.231*
	(0.0297)	(0.194)	(0.0357)	(0.0645)	(0.0431)	(0.0724)	(0.0991)
Treat	0.00763	−0.144	0.125**	0.0536	0.00157	−0.0275	−0.299*
	(0.0278)	(0.185)	(0.0385)	(0.0748)	(0.0163)	(0.0553)	(0.136)
Round	0.109***	0.678**	−0.0449	0.259***	0.0324	0.279***	0.881***
	(0.0267)	(0.180)	(0.0557)	(0.0520)	(0.0173)	(0.0682)	(0.0821)
Observations	562	562	562	562	562	562	562
R-squared	0.156	0.167					0.233
Standard errors in parentheses							
***p < 0.01, **p < 0.05, *p < 0.1							

Note: Full set of control variables, village dummies included in both models, marginal effects reported for logistic regressions.

The insignificant finding on control over assets negated one of the key pathways assumed in the theory of change of the intervention—that providing women with motor pumps would increase their control over these assets. Rather, this finding suggests that existing social norms regarding asset ownership limited the extent to which women could benefit from the intervention—qualitative research from the study sites suggests that even when women were in groups that won the lottery, these assets were turned over to and controlled by their husbands due to social norms giving men greater control over large agricultural assets, such as machinery (including pumps) and livestock (Bryan and Garner, 2020, 2022). Another explanation for the lack of significant findings on women's control over assets could be related to the fact that pumps were owned by a group of farmers rather than the household, while the asset questions in the WEAI refer to assets owned by the household. Therefore, farmers may have not perceived that they owned or had control over the pumps even if they had access to one through their group. Descriptive data support this conclusion as there was only a slight increase in the share of farmers reporting that they owned a pump between rounds 1 and 2—from 3.7% to 4.2% of men and 2.4%–3.9% of women, respectively. While women's control over assets did not increase as a result if the intervention, the results provide some evidence that overall household assets increased. The number of asset types owned increased among households who benefitted from the intervention compared to control group 2, regardless of who in the household controlled the assets.

Limited control over the pumps may also explain the fact that the intervention did not directly increase women's role in production decisions. The results also suggest that even if women's time spent irrigating declined because of the intervention, as descriptive data indicate, women likely increased time devoted to other livelihood activities. Thus, it seems that gaining access to the pump did not reduce women's overall work burden, but rather shifted time allocated to different livelihood activities.

The results also show some significant differences between women in the treatment and control groups at the baseline after controlling for other factors. Women in the treatment group already had higher individual empowerment scores and were more likely to achieve adequacy in input into agricultural production decisions. Moreover, the results indicate that women's empowerment is increasing over time. Between rounds 1 and 2, aggregate empowerment scores as well as individual adequacy scores for asset ownership and work balance increased for all women in the study areas (both treatment and control communities). This result provides evidence of an already strong trend of the growing influence of women in the study communities, irrespective of the irrigation intervention.

### 5.2 Other factors influencing women's empowerment

Other significant findings also shed light on the determinants of women's empowerment in this context. The full set of results is shown in the supplementary material, Appendix 1. The results from both models show that age is a highly important factor affecting women's overall empowerment scores as well as the likelihood that they achieve adequacy in agricultural decision-making, income decisions, and work balance, with levels of empowerment increasing with age. Difference-in-difference results show that having co-wives also generally increases women's empowerment. Using the first control group, results show that having co-wives increases the likelihood of achieving adequacy in agricultural decision-making and input into income decisions. However, results using both control groups also show a decrease in the likelihood of achieving adequacy in asset ownership with co-wives. These results suggest that having multiple wives in the household increases women's bargaining power in agriculture, especially with respect to agricultural and income decisions. Some women did, in fact, report that they were allocated plots of land by their husbands that they farmed jointly with their co-wives. It may be, therefore, that wives were more successful in accessing land and controlling the income from production when they negotiate collectively. On the other hand, asset accumulation for individual women may be more difficult when family size increases.

Women from households with larger land holdings at the baseline were less likely to have input into production decisions (both control groups), control over income (control 2), and work balance (control 1), and overall land size is associated with lower aggregate empowerment scores (control 1). On the other hand, irrigation status at the baseline is associated with higher aggregate women's empowerment scores and women's greater input into agricultural decisions (control 1) and asset ownership (both control groups). Unsurprisingly, irrigation status at baseline is associated with a higher work burden for women (control 2). This suggests that irrigation with traditional, manual methods—an activity which many women in these communities were engaged in before the intervention—already contributed to women's empowerment including women's asset accumulation, but at a cost of an increased work burden. It is also possible that women who were more empowered to begin with were more likely to engage in traditional forms of irrigation. That is, women with higher levels of agency may be able to gain access to land and water in order to participate in small-scale irrigation activities. The results also show that women are more likely to achieve adequacy in agricultural decision-making when they have access to groundwater for irrigation, compared to surface water.

Finally, climate and idiosyncratic shocks appear to influence women's empowerment outcomes. The results show that women from households who reported experiencing a climate shock in the last two years, including droughts, floods, and storms, are more likely to have higher aggregate empowerment scores (control 1) and greater participation in income decisions (both control groups). This may indicate that women contribute more of the income they earn to meeting household needs when their household experiences a climate shock and, therefore, have a larger say in income decisions. Both men and women in the study communities reported intentionally dividing income among spouses to pay for different household expenditures and to cope with shocks that may occur. Women in households that experienced an idiosyncratic shock in the last two years—defined as death or illness of a family member or theft—also have higher aggregate empowerment scores (both control groups). However, these women had less input in agricultural decision-making (both control groups), possibly due to an increased care burden, which prevented them from engaging in agriculture. These results show that women take on larger roles in the household when faced with climate and idiosyncratic shocks. They also suggest that women and men are affected differently by shocks and that women also have important roles to play in coping with idiosyncratic and covariate shocks. While women may play an important role in coping with shocks to the household, it is important to view these changes in their agency while also considering the stress that these shocks place on families. That is, while shocks may result in women taking on a larger decision-making role in some respects, such changes may reflect an increasing burden on women to maintain household well-being during times of adversity.

### 5.3 Spillover effects

Spillover effects were estimated by comparing outcomes of women in the treatment villages in households that did not win the lottery with women in control villages where no lottery was conducted. The results show that women in households that did not win the lottery were less likely to achieve adequacy in control over assets and input into income decisions than women in the control communities where no lottery was conducted even after controlling for differences between these groups at baseline and over time (Table 5). Moreover, exposure to the intervention (as represented by the number of trust groups within the larger confidence group that won the lottery) is shown to have a negative effect on women's input into agricultural production decisions.

**Table 5:** Difference-in-difference estimation of spillover effects.

	Spillover						
	A-WEAI score	No. Of adequacies	Production decisions	Ownership of assets	Income decisions	Work balance	No. Of asset types owned
SpilloverxRound	−0.0607	−0.201	−0.136	−0.205**	−0.151***	−0.0142	0.0253
	(0.0323)	(0.233)	(0.0809)	(0.0663)	(0.0306)	(0.0801)	(0.433)
Spillover	−0.00448	−0.417*	0.216**	−0.242	0.127**	0.0229	−0.345
	(0.0345)	(0.191)	(0.0670)	(0.139)	(0.0431)	(0.0551)	(0.276)
Round	0.112***	0.701***	−0.0542	0.281***	0.0485*	0.274***	0.874***
	(0.0251)	(0.170)	(0.0728)	(0.0613)	(0.0216)	(0.0662)	(0.0713)
Observations	538	538	538	538	538	538	538
R-squared	0.128	0.136					0.209
Standard errors in parentheses							
***p < 0.01, **p < 0.05, *p < 0.1							

Note: Full set of control variables, village dummies included, marginal effects reported for logistic regressions.

This somewhat surprising result suggests that the lottery may have created some conflicts in treatment villages that had a negative impact on women within households that did not win the lottery. In fact, 16 percent of lottery-winning households did report some conflicts within the community related to the pumps. Moreover, while the intervention specifically targeted communities with a relatively high potential for irrigation, water remains a scarce resource in some of the communities and some farmers reported difficulty obtaining water using the pumps in certain locations. Thus, the intervention may have increased the level of water extraction in intervention communities, whereby access to water for irrigation improved for lottery winners, while at the same time, lottery losers had reduced access to water for irrigation using manual methods. Therefore, the intervention seems to have intensified competition over resources—both the technologies and water for irrigation—leading to harmful effects on women that did not gain access to pumps and, thus, faced more difficulty in irrigated production.

## 6 Discussion

The results demonstrate that expanding access to small-scale irrigation technologies alone is not likely to increase women's agency. While women may have benefitted from increased household income and better food security, the intervention generally did not expand women's decision-making influence in key spheres of life. However, while quantitative analysis can identify general relationships and trends, it is not able to untangle the multiple and nuanced ways in which irrigation affects women in the study communities. Thus, while the quantitative results did not show significant impacts, qualitative focus group discussions and interviews with men and women farmers in the study area suggest that the impacts of the motor pump intervention are varied and may be indirect—while some women reported direct income benefits of engaging in small-scale irrigation, others described shifting time away from direct engagement in irrigation activities when motor pumps were introduced (Bryan and Garner, 2022). There also appears to be an indirect benefit of irrigation in terms of household ownership of assets, even if women themselves do not control the assets.

The lack of impact of the intervention on women's empowerment must be considered in the context of ongoing social change in the study area. The econometric results in this paper suggest that women's agency in the study areas is increasing over time with women having higher aggregate empowerment scores in both the treatment and control communities at the endline. The results are also consistent with qualitative findings that women in this region of Ghana are experiencing changes in their roles and expectations in the household and in their communities. Dramatic differences were observed between older and younger men and women in terms of educational achievements, opinions on family and household decisions, and women's role in the household. Qualitative findings indicate that women's economic contribution to their household is increasing with women contributing their own income to pay for basic household needs, like food and education expenses for children, and participating more in agricultural decisions and community leadership roles (Bryan and Garner, 2020, 2022). However, we note that the project area is one in which many development interventions are ongoing, and farmers are accustomed to dealing with external groups and receiving aid. This dynamic may have influenced the way in which farmers responded to the surveys, interviews, and focus groups.

Thus, in the context of rapid social change, the impacts of any one intervention on measures of women's empowerment is likely to be minimal, especially when the intervention does not include activities aimed at transforming the patriarchal norms that shape gender inequalities. Women in the study communities still face significant constraints to adopting and benefitting from small-scale irrigation, including limited access to productive resources given patrilineal inheritance norms, which prevent women gaining access to land (especially irrigable land) except through their husbands (Bryan and Garner, 2022; Yokying and Lambrecht, 2020). Women also lack the labor needed to dig wells and construct mud fences to protect their irrigated plots from livestock that graze openly during the dry season (Bryan and Garner, 2022). Furthermore, social norms that prohibit women from owning large assets, including the motor pumps, are a significant impediment to achieving empowerment gains from small-scale irrigation interventions. The ways in which patriarchal norms hinder women's ability to access and control assets has also been described by other research in this context (Theis et al., 2018; Lambrecht, 2016; Doss et al., 2014). Moreover, the spillover analysis revealed some negative impacts on women from households in intervention villages that did not gain access to the pumps. This suggests that when resources are scarce and access to technologies is limited, women may be at an even greater disadvantage and experience setbacks in their ability to participate in production and spending decisions and gain control over assets.

While considerable challenges to women's participation in mechanized small-scale irrigation remain, there were also several implementation challenges, including the delay in pump distribution and lack of loan repayment by some groups, that limited the benefits of the intervention for women farmers in trust groups that won the lottery. Moreover, not all households in trust groups that won the lottery had equal access to the technology. Data on perceptions of program effectiveness from the second survey round, after the intervention was implemented, indicate that the trust groups did not function as intended, with 37 percent of lottery-winning households claiming that they did not use the pump and 25 percent saying that not all trust group members had equal access to the pump. Among lottery winners, farmers who were already irrigating at the baseline were far more likely to reporting actually using the pump. This suggests that it is much more difficult for non-irrigators to use modern irrigation technologies, given additional constraints that prevent them from adopting irrigation practices in the first place. Anecdotal evidence suggests that some farmers who were able to repay the bulk of the loan had greater control over its use and distribution among group members. For all these reasons, the intent-to-treat effects may miss positive program impacts on women from households that did use the pumps for dry season irrigation.

## 7 Conclusions

The findings in this paper have important implications for the design of policies and programs aimed at expanding small-scale irrigation. Importantly, the results highlight the need for caution during the rollout of irrigation interventions given potential negative spillover effects for women in treatment communities that do not get access to the pumps and the risk of exacerbating competition over scarce water resources. In particular, randomizing the distribution of agricultural assets among households or groups within the same community may lead to conflicts and should be avoided. Rather, pumps should be made available to all farmer groups in the intervention communities to prevent possible conflicts and scaled to other communities over time. In the case of irrigation interventions, it is also important to determine whether expanding access to irrigation technologies places increased pressure on water resources leading to scarcity. If water availability for irrigation declines because of the intervention, this may exacerbate intra-community conflicts and reduce the long-term sustainability of irrigation in communities with limited water resources and infrastructure. Differences in demand for the intervention across villages, which indicate unequal access to natural resources for irrigation (i.e. land and water), suggest that distribution of motor pumps alone is not enough to spark more widespread adoption of small-scale irrigation. Other infrastructure investments, such as in dams and wells, are needed to increase water availability for irrigation in communities with adequate water resources but inadequate infrastructure to access water.

More research on alternative approaches to the diffusion of small-scale irrigation and the extent to which these facilitate women's empowerment is needed to ensure gender equity in access to and benefits from small-scale irrigation. It is important to consider the gender implications of alternative interventions to, at a minimum, do no harm to women, including non-beneficiaries. While this paper focused on one specific intervention—the distribution of motor pumps through a random lottery—other alternative interventions, by NGOs, the private sector, and other actors, are also used to expand irrigation access. The difficulties of implementing the complex intervention described in this paper suggest that other modalities of scaling small-scale irrigation should be explored further to evaluate whether they offer a more effective and inclusive option. One possible intervention is a service-based model, whereby farmers owning pump technologies could rent these to other farmers in the community and even provide labor for irrigation. Other approaches are not without their limitations—e.g. the service-based model would still require high initial investment in pump technology by the service provider, which may be especially cost-prohibitive for women. Moreover, farmers lacking access to land and water resources, including women and other resource poor farmers, might find it still too costly to access rental services. Yet some studies suggest that there are opportunities for women to benefit from mechanization services when programs are designed appropriately to ensure their participation (Theis et al., 2019).

For any small-scale irrigation intervention to benefit and empower women, it should be implemented through modalities that involve both men and women in decision-making and in project activities, such as trainings (Quisumbing et al., 2021). Another approach may be to couple irrigation projects with complementary interventions that address restrictive gender norms, like household and community dialogues that may lead to more inclusive decision-making processes about how technologies are applied and who controls the output and income from the use of those technologies (Cislaghi et al., 2019; Kumar et al., 2018; Theis et al., 2018). In addition, women's lack of access to land and water resources in the study area remains a key impediment to women being able to benefit directly from small-scale irrigation without complementary interventions that enable women to access these resources more easily.

Finally, more research is needed to weigh the irrigation pathway against alternative pathways to women's empowerment provided by other livelihood choices. Studies suggest that women's engagement in alternative livelihood activities, such as petty trading, increase their decision-making power at the household level and resilience to shocks and stresses (Lawson et al., 2020; Wrigley-Asante, 2011). Ultimately, providing multiple pathways for women's empowerment will allow women to choose the option that best meets their needs and preferences, enabling them to achieve their goals and live meaningful and fulfilling lives.

## Declaration of competing interest

The authors declare that they have no known competing financial interests or personal relationships that could have appeared to influence the work reported in this paper.

## Data Availability

Data used in the analysis will soon be available in the repository: https://dataverse.harvard.edu/dataverse/IFPRI.

## Footnotes

1 The villages of Akara and Gbanterago Alemgbek together were paired with Mognoori village; Asikiri was paired with Yidigu; Denegu was paired with Bugri Natinga; and Binipiala was paired with Zule for the random selection of villages into treatment.

2 Half of the trust groups in the randomly-selected villages were assigned into treatment through a public draw of lottery.
