# Supplementary material for: Does small-scale irrigation provide a pathway to women's empowerment? Lessons from Northern Ghana
Source: J Rural Stud. 2023 Jan;97:474–84. doi: 10.1016/j.jrurstud.2022.12.035 (PMC9930902; doi:10.1016/j.jrurstud.2022.12.035)
Supplement: Multimedia component 1 [file mmc1.docx]

**Appendix 1: Difference in Difference Estimations, Full Results Tables**

Appendix Table 1.1: Difference-in-Difference Intent to Treat Effect on Indicators of Women’s Empowerment, Control Group 1, Full Results Table

|  | Control 1 | | | | | |  |
| --- | --- | --- | --- | --- | --- | --- | --- |
|  | A-WEAI score | No. of adequacies | Production decisions | Ownership of assets | Income decisions | Work balance | No. of asset types owned |
| TreatxRound | 0.00124 | 0.0999 | -0.00467 | 0.145 | 0.0700 | -0.0805 | 0.258 |
|  | (0.0158) | (0.100) | (0.0453) | (0.0792) | (0.0428) | (0.0473) | (0.221) |
| Treat | 0.0507*** | 0.252** | 0.0807*** | 0.0197 | 0.0109 | 0.0308 | -0.439** |
|  | (0.0125) | (0.0697) | (0.0198) | (0.0433) | (0.0489) | (0.0434) | (0.151) |
| Round | 0.0904** | 0.624*** | -0.0904 | 0.198** | 0.00880 | 0.270*** | 0.894*** |
|  | (0.0244) | (0.124) | (0.0686) | (0.0499) | (0.0268) | (0.0403) | (0.196) |
| Age | 0.00296*** | 0.0161*** | 0.00559*** | 0.00340 | 0.00235*** | 0.00350 | -0.0201*** |
|  | (0.000435) | (0.00219) | (0.000496) | (0.00188) | (0.000219) | (0.00236) | (0.00322) |
| Schooling | -0.0145 | -0.0382 | 0.0365 | -0.0430 | 0.00198 | -0.0756 | 0.431 |
|  | (0.0310) | (0.170) | (0.0320) | (0.0622) | (0.0309) | (0.0708) | (0.241) |
| Cowives | 0.0204 | 0.111 | 0.0589* | -0.0551* | 0.0300** | 0.00669 | 0.0619 |
|  | (0.0148) | (0.0857) | (0.0243) | (0.0260) | (0.00961) | (0.0241) | (0.223) |
| Muslim | -0.00708 | -0.0339 | 0.0255 | 0.00460 | 0.0267 | -0.00804 | 0.192 |
|  | (0.0249) | (0.134) | (0.0217) | (0.0555) | (0.0145) | (0.0681) | (0.232) |
| Traditional | -0.0268 | -0.113 | -0.00797 | 0.0498* | -0.0117 | -0.0348 | 0.00529 |
|  | (0.0162) | (0.0874) | (0.0207) | (0.0217) | (0.0212) | (0.0625) | (0.330) |
| Household size | -0.000589 | -0.0121 | -0.000777 | -0.00762 | -0.00136 | 0.00327 | 0.118** |
|  | (0.00112) | (0.00747) | (0.00272) | (0.0113) | (0.00297) | (0.00557) | (0.0435) |
| Children under 5 | -0.00176 | 0.0168 | 0.00467 | 0.0137 | 0.00483 | -0.0198 | -0.212 |
|  | (0.00648) | (0.0341) | (0.0135) | (0.0194) | (0.00882) | (0.0142) | (0.138) |
| Land size at baseline | -0.00264** | -0.00996** | -0.00609** | 0.00134 | -0.00112 | -0.00828* | -0.0183 |
|  | (0.000972) | (0.00312) | (0.00166) | (0.00403) | (0.00141) | (0.00375) | (0.0329) |
| Plot distance | 9.43e-06 | 0.000932 | -0.000995 | 0.000669 | -1.96e-05 | -0.000709 | 0.00560 |
|  | (0.000586) | (0.00292) | (0.000687) | (0.000776) | (0.000299) | (0.00133) | (0.00509) |
| TLU at baseline | -0.00162 | -0.00755 | -0.00387 | -0.00304* | -0.000170 | -0.000918 | 0.0785** |
|  | (0.00190) | (0.00892) | (0.00360) | (0.00122) | (0.00219) | (0.000842) | (0.0253) |
| Irrigation at baseline | 0.0394** | 0.224* | 0.0573* | 0.0787** | 0.0178 | -0.00792 | 0.191 |
|  | (0.0144) | (0.0875) | (0.0281) | (0.0264) | (0.0153) | (0.0315) | (0.131) |
| Water source | -0.0124 | -0.104 | -0.0733** | 0.0424 | 0.00176 | 0.0310 | 0.497 |
|  | (0.0103) | (0.0567) | (0.0201) | (0.0777) | (0.0238) | (0.0347) | (0.456) |
| Climate shock | 0.0292* | 0.161* | 0.0303 | 0.0268 | 0.0453* | 0.000494 | -0.113 |
|  | (0.0129) | (0.0787) | (0.0264) | (0.0436) | (0.0189) | (0.0387) | (0.124) |
| Idiosyncratic shock | 0.0307* | 0.225** | -0.0675* | 0.0587 | 0.0307 | 0.00878 | 0.564*** |
|  | (0.0146) | (0.0811) | (0.0327) | (0.0412) | (0.0204) | (0.0176) | (0.126) |
| Constant | 0.526*** | 3.131*** |  |  |  |  | 5.482*** |
|  | (0.0456) | (0.262) |  |  |  |  | (0.280) |
| Observations | 770 | 770 | 770 | 770 | 770 | 770 | 770 |
| R-squared | 0.140 | 0.156 |  |  |  |  | 0.198 |
| Standard errors in parentheses | |  |  |  |  |  |  |
| *** p<0.01, ** p<0.05, * p<0.1 | |  |  |  |  |  |  |
| Note: Village dummies included in all models, marginal effects reported for logistic regressions | | | | | | |  |

Appendix Table 1.2: Difference-in-Difference Intent to Treat Effect on Indicators of Women’s Empowerment, Control Group 2, Full Results Table

|  | Control 2 | | | | | | |
| --- | --- | --- | --- | --- | --- | --- | --- |
|  | A-WEAI score | No. of adequacies | Production decisions | Ownership of assets | Income decisions | Work balance | No. of asset types owned |
| TreatxRound | -0.0189 | 0.0356 | -0.0445 | 0.0552 | 0.0353 | -0.0858 | 0.231* |
|  | (0.0297) | (0.194) | (0.0357) | (0.0645) | (0.0431) | (0.0724) | (0.0991) |
| Treat | 0.00763 | -0.144 | 0.125** | 0.0536 | 0.00157 | -0.0275 | -0.299* |
|  | (0.0278) | (0.185) | (0.0385) | (0.0748) | (0.0163) | (0.0553) | (0.136) |
| Round | 0.109*** | 0.678** | -0.0449 | 0.259*** | 0.0324 | 0.279*** | 0.881*** |
|  | (0.0267) | (0.180) | (0.0557) | (0.0520) | (0.0173) | (0.0682) | (0.0821) |
| Age | 0.00253*** | 0.0143*** | 0.00532*** | 0.00198 | 0.00152*** | 0.00146 | -0.0230*** |
|  | (0.000539) | (0.00200) | (0.000673) | (0.00174) | (0.000371) | (0.00172) | (0.00315) |
| Schooling | -0.0223 | -0.0730 | 0.0261 | -0.0639 | -0.0154 | -0.104 | 0.388 |
|  | (0.0275) | (0.139) | (0.0366) | (0.0617) | (0.0165) | (0.0927) | (0.208) |
| Cowives | 0.0232 | 0.133 | 0.0499 | -0.100*** | 0.0154 | 0.0402* | 0.0505 |
|  | (0.0170) | (0.0874) | (0.0368) | (0.0187) | (0.00949) | (0.0170) | (0.302) |
| Muslim | -0.0240 | -0.107 | -0.00273 | -0.0333 | 0.00805 | -0.0432 | 0.0519 |
|  | (0.0205) | (0.0912) | (0.0441) | (0.0305) | (0.0196) | (0.0686) | (0.154) |
| Traditional | -0.0355* | -0.122 | -0.0327 | 0.0503 | -0.0203 | -0.0671 | 0.124 |
|  | (0.0157) | (0.0963) | (0.0318) | (0.0368) | (0.0185) | (0.0932) | (0.419) |
| Household size | 0.000578 | -0.00414 | -0.00240 | 0.00128 | -0.00148 | 0.000191 | 0.140*** |
|  | (0.00175) | (0.00872) | (0.00292) | (0.0117) | (0.00268) | (0.00549) | (0.0330) |
| Children under 5 | -0.00826 | -0.0226 | 0.00263 | -0.00461 | 0.000727 | -0.0236 | -0.288* |
|  | (0.00584) | (0.0343) | (0.0204) | (0.0145) | (0.00773) | (0.0144) | (0.116) |
| Land size at baseline | -0.00263 | -0.0118 | -0.00444** | -0.00369 | -0.00167* | -0.00482 | -0.0400 |
|  | (0.00163) | (0.00779) | (0.00131) | (0.00392) | (0.000739) | (0.00497) | (0.0424) |
| Plot distance | 4.52e-06 | 0.000749 | -0.00147 | 0.000735 | -0.000151 | -0.000141 | 0.00726 |
|  | (0.000654) | (0.00343) | (0.000781) | (0.000600) | (0.000256) | (0.00199) | (0.00617) |
| TLU at baseline | -0.00106 | -0.00519 | -0.00182 | -0.00270 | 0.00134 | -0.00163* | 0.0678** |
|  | (0.00173) | (0.00886) | (0.00276) | (0.00171) | (0.00136) | (0.000642) | (0.0216) |
| Irrigation at baseline | 0.0390 | 0.242 | 0.0406 | 0.0929* | 0.0182 | -0.0489** | 0.285 |
|  | (0.0228) | (0.138) | (0.0366) | (0.0410) | (0.0159) | (0.0155) | (0.206) |
| Water source | -0.0192 | -0.143 | -0.0729*** | 0.0192 | -0.00947 | 0.0329 | 0.714 |
|  | (0.0114) | (0.0767) | (0.0169) | (0.103) | (0.0198) | (0.0554) | (0.384) |
| Climate shock | 0.0119 | 0.0544 | 0.00993 | 0.0300 | 0.0287** | 0.00173 | 0.0569 |
|  | (0.00839) | (0.0723) | (0.0130) | (0.0646) | (0.00912) | (0.0465) | (0.126) |
| Idiosyncratic shock | 0.0250 | 0.213* | -0.0934** | 0.0723 | 0.0138 | 0.00976 | 0.633** |
|  | (0.0160) | (0.0999) | (0.0301) | (0.0593) | (0.0151) | (0.0232) | (0.179) |
| Constant | 0.554*** | 3.252*** |  |  |  |  | 5.521*** |
|  | (0.0533) | (0.185) |  |  |  |  | (0.256) |
| Observations | 562 | 562 | 562 | 562 | 562 | 562 | 562 |
| R-squared | 0.156 | 0.167 |  |  |  |  | 0.233 |
| Standard errors in parentheses | |  |  |  |  |  |  |
| *** p<0.01, ** p<0.05, * p<0.1 | |  |  |  |  |  |  |
| Note: Village dummies included in all models, marginal effects reported for logistic regressions | | | | | | |  |

Appendix Table 1.3: Difference-in-Difference Estimation of Spillover Effects, Full Results Table

|  | A-WEAI score | No. of adequacies | Production decisions | Ownership of assets | Income decisions | Work balance | No. of asset types owned |
| --- | --- | --- | --- | --- | --- | --- | --- |
| SpilloverxRound | -0.0607 | -0.201 | -0.136 | -0.205** | -0.151*** | -0.0142 | 0.0253 |
|  | (0.0323) | (0.233) | (0.0809) | (0.0663) | (0.0306) | (0.0801) | (0.433) |
| Spillover | -0.00448 | -0.417* | 0.216** | -0.242 | 0.127** | 0.0229 | -0.345 |
|  | (0.0345) | (0.191) | (0.0670) | (0.139) | (0.0431) | (0.0551) | (0.276) |
| Round | 0.112*** | 0.701*** | -0.0542 | 0.281*** | 0.0485* | 0.274*** | 0.874*** |
|  | (0.0251) | (0.170) | (0.0728) | (0.0613) | (0.0216) | (0.0662) | (0.0713) |
| Intensity of treatment | -0.00676 | -0.0209 | -0.0441* | 0.0306 | -0.00781 | 0.000219 | -0.122* |
|  | (0.0130) | (0.0663) | (0.0211) | (0.0172) | (0.00500) | (0.0330) | (0.0562) |
| Age | 0.00319*** | 0.0169*** | 0.00542*** | 0.00406* | 0.00300*** | 0.00365 | -0.0217** |
|  | (0.000642) | (0.00320) | (0.000942) | (0.00193) | (0.000416) | (0.00288) | (0.00666) |
| Schooling | -0.0332 | -0.0998 | -0.0229 | -0.0391 | -0.0322 | -0.0218 | 0.445 |
|  | (0.0394) | (0.229) | (0.0284) | (0.0855) | (0.0278) | (0.0666) | (0.279) |
| Cowives | 0.0323 | 0.178 | 0.0930** | -0.0501 | 0.0376* | -0.000788 | 0.166 |
|  | (0.0174) | (0.0948) | (0.0301) | (0.0515) | (0.0169) | (0.0420) | (0.297) |
| Muslim | -0.00293 | -0.0301 | 0.0637* | 0.0122 | 0.0483** | -0.0144 | 0.0431 |
|  | (0.0323) | (0.162) | (0.0314) | (0.0701) | (0.0161) | (0.0755) | (0.269) |
| Traditional | -0.0235 | -0.115 | 0.0239 | 0.0703* | -0.00508 | 0.0159 | -0.229 |
|  | (0.0182) | (0.101) | (0.0312) | (0.0295) | (0.0248) | (0.0839) | (0.244) |
| Household size | -0.000909 | -0.0161 | 0.00118 | -0.00271 | 0.00162 | 0.00279 | 0.139** |
|  | (0.00180) | (0.0113) | (0.00384) | (0.0137) | (0.00240) | (0.00916) | (0.0462) |
| Children under 5 | -0.00124 | 0.0420 | -0.00980 | 0.0382 | 0.00374 | -0.0250 | -0.234 |
|  | (0.0107) | (0.0730) | (0.0151) | (0.0306) | (0.0133) | (0.0164) | (0.166) |
| Land size at baseline | -0.00223 | -0.00663 | -0.00889*** | 0.000353 | -0.00187 | -0.00881 | -0.0487 |
|  | (0.00155) | (0.00464) | (0.00219) | (0.00617) | (0.00255) | (0.00812) | (0.0357) |
| Plot distance | 1.73e-05 | 0.000795 | -0.00144 | 0.00118 | 1.09e-05 | -0.000785 | 0.00353 |
|  | (0.000763) | (0.00391) | (0.00106) | (0.00155) | (0.000437) | (0.00177) | (0.00893) |
| TLU at baseline | -0.00120 | -0.00569 | -0.00337 | -0.00340** | 0.00106 | -0.000527 | 0.0686** |
|  | (0.00162) | (0.00730) | (0.00283) | (0.00118) | (0.00238) | (0.00135) | (0.0232) |
| Irrigation at baseline | 0.0399 | 0.202 | 0.0852* | 0.0994** | 0.0349 | -0.00467 | 0.239 |
|  | (0.0205) | (0.121) | (0.0358) | (0.0294) | (0.0195) | (0.0588) | (0.134) |
| Water source | -0.0135 | -0.0603 | -0.0920** | 0.108 | -0.0126 | 0.0417 | 0.762 |
|  | (0.0157) | (0.0694) | (0.0323) | (0.0568) | (0.0332) | (0.0598) | (0.461) |
| Climate shock | 0.0395 | 0.226 | 0.0377 | 0.0140 | 0.0592* | -0.0378 | -0.163 |
|  | (0.0219) | (0.136) | (0.0307) | (0.0469) | (0.0231) | (0.0257) | (0.182) |
| Idiosyncratic shock | 0.0241 | 0.185* | -0.0841* | 0.0205 | 0.0329 | -0.00296 | 0.582** |
|  | (0.0159) | (0.0844) | (0.0350) | (0.0433) | (0.0253) | (0.0166) | (0.172) |
| Constant | 0.497*** | 3.027*** |  |  |  |  | 5.844*** |
|  | (0.0752) | (0.408) |  |  |  |  | (0.440) |
| Observations | 538 | 538 | 538 | 538 | 538 | 538 | 538 |
| R-squared | 0.128 | 0.136 |  |  |  |  | 0.209 |
| Standard errors in parentheses | |  |  |  |  |  |  |
| *** p<0.01, ** p<0.05, * p<0.1 | |  |  |  |  |  |  |
| Note: Village dummies included, marginal effects reported for logistic regressions | | | | |  |  |  |

Appendix Table 1.4: Balance Tests between spillover and control group 2

| Dependent vars | Spillover (obs=139) | Control2 (obs=238) |  |
| --- | --- | --- | --- |
| 5DE score | 0.71 | 0.68 |  |
| Number of adequacies | 4 | 3.97 |  |
| Input into agricultural decisions | 0.9 | 0.82 | * |
| Asset ownership | 0.59 | 0.54 |  |
| Control over income | 0.95 | 0.86 | ** |
| Work balance | 0.58 | 0.49 | * |
| Number of asset types owned | 5.85 | 5.84 |  |
