# Supplementary material for: Does small-scale irrigation provide a pathway to women's empowerment? Lessons from Northern Ghana
Source: J Rural Stud. 2023 Jan;97:474–84. doi: 10.1016/j.jrurstud.2022.12.035 (PMC9930902; doi:10.1016/j.jrurstud.2022.12.035)
Supplement: Multimedia component 2 [file mmc2.docx]

**Appendix 2: Single Difference Estimation and Results**

*A.2.1 Methods and Data*

The motor pumps for small-scale irrigation were randomly distributed to farmer groups. While we prefer the difference-in-difference results presented in the main manuscript, given that it controls for baseline differences between treatment and control communities and changes over time; assuming the randomization was successful, a first difference model may be estimated to measure the impact of the intervention on indicators of women’s empowerment. We present the results of the following model in this appendix:

Y_i_ = β_0_ + β_1_T_i_ + β_x_X_i_ + ɛ

Where B_i_ captures the effect of the treatment on the different indicators of women’s empowerment (Y_i_): the individual Pro-WEAI score (3DE), number of adequacies (0-10), individual adequacy scores for input into production decisions, control over assets, control over income, and work balance, and X represents the set of control variables. As with the difference-in difference results, this model is applied to estimate the impact of the treatment using both control groups and to estimate spillover effects in the treated communities.

One key difference between the first difference and difference-in-difference models is that they use different aggregate measures of women’s empowerment. During the endline survey round, additional modules were added to the intra-household survey as part of a pilot test of the project-level Women’s Empowerment in Agriculture Index (pro-WEAI). These included modules on mobility, attitudes about domestic violence, membership in influential groups, and self-efficacy.

The pro-WEAI contains 12 sub-indicators, which are mapped to 3 domains of empowerment: intrinsic, instrumental, and collective agency (Malapit et al. 2019). The version of the pro-WEAI used in this paper is comprised of 10 sub-indicators all given equal weight in the index.^[[1]](#footnote-1)^ These indicators are shown in Appendix Table 2.1 with the definition of adequacy for each indicator. Six of these indicators are shared with the A-WEAI and these indicators are highlighted in bold. Thus, the aggregate pro-WEAI measures—the individual pro-WEAI score (10 indicators) and the number of adequacies (out of 10) are used in the single difference model that relies on endline data. The single difference model was run also for the same sub-indicators as in the difference-in-difference model: input into productive decisions, ownership of assets, control over income, and work balance. As with the difference-in-difference model, we include an outcome variable for number of asset categories owned by the household to explore whether the intervention had overall benefits for the household.

Appendix Table 2.1: Pro-WEAI Domains, Indicators, and Definitions

| **Domain** | **Indicator** | **Definition of Adequacy** |
| --- | --- | --- |
| Intrinsic agency | Self-efficacy | “Agree” or “strongly agree” with a set of self-efficacy questions |
|  | Attitudes about intimate partner violence | Believes husband is NOT justified in hitting or beating his wife in all 5 different scenarios. |
| Instrumental Agency | **Input into productive decisions** | **Makes decisions solely, jointly (and feels they have at least some input into decisions), or feels they could make the decision if they wanted to for a range of production decisions.** |
|  | **Ownership of land and other assets** | **Owns solely or jointly at least one of the following: three small assets or land.** |
|  | **Access to and decisions on credit** | **Belongs to a household that used credit in the past year and participated in at least one decision about it OR feels they could access credit if they wanted to.** |
|  | **Control over the use of income** | **Has input in decisions about how to use income and output of all agricultural and non-agricultural activities they participate in.** |
|  | **Work balance** | **Works less than 10.5 h per day:**  **Workload = time spent in primary activity + (1/2) time spent in childcare as a secondary activity** |
|  | Visiting important locations | Visits at least two locations once per week (city/market/relatives) OR visits one location at least one per month (health facility/public meeting) |
| Collective agency | **Group membership** | **Active member of at least one group** |
|  | Membership in influential groups | Active member of at least one group that can influence the community to a medium extent or greater. |

Source: Adapted from Malapit et al. (2019)

Note: Variables in bold are those that comprise the A-WEAI.

*A.2.2 First Difference Results*

While the difference-in-difference results showed no significant program impacts after controlling for differences between treatment and control groups at the baseline and trends over time, first-difference results using control group 1 show that women in households that won the lottery for a motor pump had higher aggregate empowerment scores (pro-WEAI score and number of adequacies) and were more likely to achieve adequacy in control over assets (Appendix Table 2.2). However, the results also show that the introduction of motor pumps increased women’s work burden, with women in the treatment group less likely to achieve adequacy in work balance compared to control group 1.

While the results using the first control group show positive effects on women’s empowerment, the results using the second control group (women in the control communities only) show conflicting results. Using control group 2, women in households that won the lottery are shown to have lower aggregate empowerment scores and less control over income decisions. Moreover, results using this control group suggest the intervention reduced the number of asset types owned by the household. However, the results using control group 2 also suggest the program had positive impacts on women’s involvement in production decisions and control over assets.

Overall, these results underscore that the introduction of motor pumps affected aspects of women’s empowerment in different ways leading to conflicting results using the aggregate measures of empowerment. While motor pumps may have increased women’s control over assets and women’s role in agricultural decisions, it also increased women’s work burden and reduced their control over income. The positive results of the intervention shown here suggest that positive social trends in the study communities were accelerated by the introduction of new agricultural technologies. In particular, women’s role in agricultural decision-making has been increasing over time in the study communities and women have been able to increase their access to and control over assets through participation in groups and greater income earning opportunities, often outside of agriculture (Bryan and Garner 2020). However, these results also suggest some downsides of small-scale irrigation for women. In general, engaging in irrigated production increases the workload of farmers, particularly in the dry season, when farmers engage in irrigated cultivation. Descriptive analysis of our plot level survey data shows that women and men both provide labor for irrigation using traditional methods, with women spending more time on irrigation activities on average. When motor pumps are used, men are more likely to provide labor for irrigation, however, women still spend more time irrigating, on average, when involved. Evidence from qualitative research similarly suggests that when motor pumps are introduced, men take over irrigation activities, freeing up women’s time to engage in other livelihood activities (Bryan and Garner 2020). Thus, the results that women’s workloads increase with the introduction of motor pumps may be indirect—with women spending more time on other income-earning activities. The result on control over income suggests that men may have more control over income following the introduction of motorized pumps. This result is plausible—it suggests that men control more of the income from the sale of irrigated crops when motor pumps are used for irrigation, compared to when women irrigate using traditional methods.

Consistent with the difference-in-difference model, the first difference results show mostly negative spillover effects for women in treatment villages that did not win access to the motor pumps through the lottery (Appendix Table 2.3). Results show that there were negative spillover effects with respect to aggregate empowerment scores and women’s control over assets. There were also negative effects regarding ownership of household assets. This could be because women in the treatment villages anticipated receiving later benefits from the program and decided to forgo purchasing assets, while women in control villages may have been more inclined to invest in productive assets. It may also relate to challenges women who did not have access to the pumps faced in accessing water for irrigation in communities where more water was being extracted (because of the lottery winners use of the pumps). However, the results also suggest some positive spillover benefits of the program in terms of women’s participation in agricultural decisions. That is, even women whose households did not receive pumps in villages where the lottery was conducted saw an increase in their role in production decisions.

As with the difference-in-difference results, other factors emerge as important determinants of women’s empowerment in this context. First, the results show that empowerment increases with age. Similarly, women with small children under 5 are less likely to be empowered (aggregate scores). Not surprisingly, larger land size is associated with an increasing work burden for women (both control groups). Climate and idiosyncratic shocks appear to influence women’s empowerment outcomes. First difference results show climate shocks as having a negative impact on both aggregate empowerment scores (number of adequacies) and control over income (control group 2), with evidence suggesting that these results are driven by negative effects on women’s self-efficacy and attitudes towards domestic violence (results not shown). Women in households that experienced an idiosyncratic shock—defined as death or illness of a family member or theft—have higher aggregate empowerment scores, suggesting that women take on larger roles in the household when faced with idiosyncratic shocks.

Appendix Table 2.2a: First Difference Results, Control 1

|  | Control 1 | | | | | |  |
| --- | --- | --- | --- | --- | --- | --- | --- |
|  | Pro-WEAI score | No. of adequacies | Production decisions | Ownership of assets | Income decisions | Work balance | No. of asset types owned |
| Treat | 0.0241** | 0.242** | 0.0799 | 0.138* | 0.0283 | -0.0678* | -0.0206 |
|  | (0.00655) | (0.0654) | (0.0585) | (0.0594) | (0.0589) | (0.0282) | (0.281) |
| Age | 0.000179 | 0.00182 | 0.00577** | 0.00140* | 0.00343 | 0.00487*** | -0.0218*** |
|  | (0.000311) | (0.00313) | (0.00183) | (0.000684) | (0.00240) | (0.00114) | (0.00424) |
| Schooling | 0.0305 | 0.298 | 0.0441 | -0.0114 | -0.0281 | 0.00222 | -0.0214 |
|  | (0.0296) | (0.296) | (0.0521) | (0.0394) | (0.0541) | (0.0799) | (0.141) |
| Cowives | -0.0102 | -0.0961 | 0.0407 | -0.0470 | -0.00914 | -0.0675** | 0.0637 |
|  | (0.0105) | (0.108) | (0.0220) | (0.0404) | (0.0310) | (0.0179) | (0.148) |
| Muslim | -0.0228 | -0.243 | 0.0209 | -0.0316 | -0.0162 | 0.0160 | -0.0135 |
|  | (0.0186) | (0.185) | (0.0185) | (0.0418) | (0.0284) | (0.0520) | (0.176) |
| Traditional | -0.0518 | -0.532 | 0.0606 | 0.0602** | -0.0460 | -0.0518 | -0.150 |
|  | (0.0291) | (0.282) | (0.0493) | (0.0190) | (0.0983) | (0.0824) | (0.410) |
| Household size | -0.00177 | -0.0141 | -0.00983 | -0.00229 | 0.0119** | -0.000535 | 0.0926** |
|  | (0.00260) | (0.0230) | (0.00696) | (0.00693) | (0.00458) | (0.00426) | (0.0349) |
| Children under 5 | -0.0136** | -0.137** | 0.0457 | 0.0299 | -0.00792 | -0.0114 | -0.0613 |
|  | (0.00523) | (0.0528) | (0.0266) | (0.0188) | (0.0243) | (0.0124) | (0.0994) |
| Land size | -0.00230 | -0.0231 | -0.00104 | -0.00196 | -0.00293 | -0.00807** | 0.0238* |
|  | (0.00165) | (0.0165) | (0.00242) | (0.00232) | (0.00273) | (0.00235) | (0.00987) |
| Plot distance | 0.000769 | 0.00770 | 0.000769 | 0.00113 | 0.00314** | 0.000180 | 0.00934 |
|  | (0.000462) | (0.00465) | (0.000586) | (0.00106) | (0.000794) | (0.00193) | (0.00702) |
| TLU | 0.000719 | 0.00699 | -0.00474 | -0.00393 | -0.00538 | 0.00979* | 0.121** |
|  | (0.00293) | (0.0288) | (0.00278) | (0.00261) | (0.00380) | (0.00404) | (0.0367) |
| Irrigation at baseline | 0.0121 | 0.130 | 0.0175 | 0.00256 | -0.0420 | -0.0491 | 0.832*** |
|  | (0.0212) | (0.209) | (0.0614) | (0.0445) | (0.0410) | (0.0850) | (0.183) |
| Water source | -0.0163 | -0.165 | -0.0621 | 0.00667 | -0.0115 | 0.0629 | -0.118 |
|  | (0.0161) | (0.161) | (0.0581) | (0.0685) | (0.0716) | (0.0720) | (0.287) |
| Climate shock | -0.0188 | -0.180 | 0.0300 | -0.00916 | -0.0106 | 0.0140 | -0.0819 |
|  | (0.0272) | (0.264) | (0.0457) | (0.0498) | (0.0568) | (0.0306) | (0.112) |
| Idiosyncratic shock | 0.0561*** | 0.558*** | -0.0506 | 0.0174 | -0.0451 | -0.0158 | 0.748** |
|  | (0.00921) | (0.0893) | (0.0431) | (0.0659) | (0.0495) | (0.0396) | (0.205) |
| Constant | 0.693*** | 6.926*** |  |  |  |  | 5.890*** |
|  | (0.0113) | (0.111) |  |  |  |  | (0.354) |
| Observations | 522 | 522 | 522 | 522 | 522 | 522 | 522 |
| R-squared | 0.099 | 0.099 |  |  |  |  | 0.277 |
| Robust standard errors in parentheses | |  |  |  |  |  |  |
| *** p<0.01, ** p<0.05, * p<0.1 | |  |  |  |  |  |  |
| Note: Village dummies included, marginal effects reported for logistic regressions | | | | |  |  |  |

Appendix Table 2.2b: First Difference Results, Control 2

|  | Control 2 | | | | | | |
| --- | --- | --- | --- | --- | --- | --- | --- |
|  | Pro-WEAI score | No. of adequacies | Production decisions | Ownership of assets | Income decisions | Work balance | No. of asset types owned |
| Treat | -0.0338** | -0.371** | 0.0696** | 0.172** | -0.179*** | -0.194 | -0.643* |
|  | (0.00955) | (0.0927) | (0.0202) | (0.0558) | (0.0307) | (0.0974) | (0.253) |
| Age | 0.000641* | 0.00645* | 0.00562** | 0.00100 | 0.00271 | 0.00409* | -0.0216*** |
|  | (0.000288) | (0.00276) | (0.00216) | (0.000780) | (0.00226) | (0.00161) | (0.00527) |
| Schooling | 0.0402 | 0.394 | 0.0217 | -0.0369 | -0.0461 | -0.00129 | 0.0421 |
|  | (0.0308) | (0.313) | (0.0578) | (0.0363) | (0.0499) | (0.0903) | (0.190) |
| Cowives | -0.00328 | -0.0240 | 0.0416 | -0.0295 | -0.0207 | -0.0685** | 0.0148 |
|  | (0.00642) | (0.0613) | (0.0308) | (0.0323) | (0.0294) | (0.0206) | (0.185) |
| Muslim | -0.0357* | -0.377** | -0.0278 | -0.0466 | -0.0666 | -0.0183 | -0.104 |
|  | (0.0157) | (0.144) | (0.0627) | (0.0529) | (0.0401) | (0.0590) | (0.154) |
| Traditional | -0.0519 | -0.536 | 0.0213 | 0.0736 | -0.117 | -0.0704 | -0.105 |
|  | (0.0333) | (0.323) | (0.0576) | (0.0462) | (0.0997) | (0.0991) | (0.649) |
| Household size | -0.00108 | -0.00553 | -0.0129 | 0.00123 | 0.0143 | -0.00788* | 0.0997* |
|  | (0.00358) | (0.0312) | (0.00765) | (0.00465) | (0.00708) | (0.00386) | (0.0419) |
| Children under 5 | -0.00962* | -0.0982* | 0.0605* | 0.0202 | -0.0226 | -0.00629 | -0.0603 |
|  | (0.00410) | (0.0404) | (0.0296) | (0.0212) | (0.0270) | (0.0155) | (0.142) |
| Land size | -0.00365 | -0.0366 | -0.00333 | -0.00436 | -0.00423 | -0.00803*** | 0.0136 |
|  | (0.00205) | (0.0204) | (0.00216) | (0.00281) | (0.00270) | (0.000733) | (0.00700) |
| Plot distance | 0.00103 | 0.0102 | 3.95e-05 | 0.000635 | 0.00321** | 0.00127 | 0.00447 |
|  | (0.000638) | (0.00636) | (0.000656) | (0.00142) | (0.000912) | (0.00300) | (0.0111) |
| TLU | 0.00315 | 0.0309 | 0.000641 | -0.00139 | 2.43e-05 | 0.0104* | 0.144** |
|  | (0.00338) | (0.0329) | (0.00263) | (0.00254) | (0.00235) | (0.00507) | (0.0444) |
| Irrigation at baseline | -0.00367 | -0.0237 | 0.00812 | 0.0296 | -0.0217 | -0.0868 | 0.736** |
|  | (0.0158) | (0.157) | (0.0529) | (0.0564) | (0.0385) | (0.0974) | (0.256) |
| Water source | -0.0294 | -0.300 | -0.0770 | -0.0683 | -0.0440 | 0.0476 | 0.0379 |
|  | (0.0243) | (0.245) | (0.0434) | (0.0648) | (0.0766) | (0.0989) | (0.285) |
| Climate shock | -0.0487 | -0.476* | -0.0111 | -0.0428 | -0.0769** | 0.0201 | -0.0826 |
|  | (0.0243) | (0.233) | (0.0246) | (0.0585) | (0.0271) | (0.0608) | (0.154) |
| Idiosyncratic shock | 0.0613*** | 0.608*** | -0.0499 | -0.00117 | -0.0345 | -0.0262 | 0.695** |
|  | (0.0145) | (0.143) | (0.0514) | (0.0684) | (0.0701) | (0.0570) | (0.260) |
| Constant | 0.680*** | 6.782*** |  |  |  |  | 6.038*** |
|  | (0.0317) | (0.293) |  |  |  |  | (0.487) |
| Observations | 375 | 375 |  |  |  |  | 375 |
| R-squared | 0.144 | 0.143 |  |  |  |  | 0.274 |
| Robust standard errors in parentheses | | |  |  |  |  |  |
| *** p<0.01, ** p<0.05, * p<0.1 | |  |  |  |  |  |  |
| Note: Village dummies included, marginal effects reported for logistic regressions | | | | | |  |  |

Appendix Table 2.3: First Difference, Spillover Effects

|  | Pro-WEAI score | No. of adequacies | Production decisions | Ownership of assets | Income decisions | Work balance | No. of asset types owned |
| --- | --- | --- | --- | --- | --- | --- | --- |
| Spillover | -0.0626* | -0.665** | 0.125* | -0.215** | -0.0721 | -0.0521 | -1.913*** |
|  | (0.0262) | (0.251) | (0.0488) | (0.0654) | (0.0457) | (0.0546) | (0.202) |
| Intensity of treatment | -0.0115* | -0.119* | -0.0148 | 0.00473 | -0.0106 | -0.00345 | -0.0716 |
|  | (0.00472) | (0.0477) | (0.0279) | (0.0129) | (0.0281) | (0.00584) | (0.0624) |
| Age | -0.000101 | -0.000982 | 0.00471* | 0.00201*** | 0.00265 | 0.00471** | -0.0214*** |
|  | (0.000520) | (0.00520) | (0.00207) | (0.000493) | (0.00248) | (0.00160) | (0.00355) |
| Schooling | 0.0431 | 0.422 | -0.0392 | -0.0356 | -0.0642 | 0.0451 | -0.0778 |
|  | (0.0357) | (0.359) | (0.0455) | (0.0328) | (0.0493) | (0.0830) | (0.187) |
| Cowives | -0.00620 | -0.0535 | 0.0457 | -0.0433 | 0.00593 | -0.0580** | 0.206 |
|  | (0.00868) | (0.0889) | (0.0307) | (0.0380) | (0.0265) | (0.0184) | (0.185) |
| Muslim | -0.0233 | -0.252 | 0.0730* | -0.0172 | 0.00738 | 0.0399 | -0.138 |
|  | (0.0236) | (0.230) | (0.0350) | (0.0600) | (0.0539) | (0.0641) | (0.154) |
| Traditional | -0.0623 | -0.642 | 0.0693 | 0.0551 | -0.0687 | -0.0500 | -0.488 |
|  | (0.0381) | (0.368) | (0.0556) | (0.0524) | (0.107) | (0.0935) | (0.254) |
| Household size | -0.00233 | -0.0185 | -0.00859 | -0.00201 | 0.0135* | 0.00427 | 0.0867* |
|  | (0.00395) | (0.0360) | (0.0100) | (0.00731) | (0.00639) | (0.00925) | (0.0369) |
| Children under 5 | -0.0169* | -0.170* | 0.0372 | 0.0361 | 0.00297 | -0.0251 | -0.157 |
|  | (0.00740) | (0.0744) | (0.0357) | (0.0238) | (0.0240) | (0.0170) | (0.0994) |
| Land size | -0.00339 | -0.0340 | -0.00157 | -0.00210 | -0.00643** | -0.00740* | 0.0314* |
|  | (0.00194) | (0.0193) | (0.00310) | (0.00319) | (0.00215) | (0.00309) | (0.0128) |
| Plot distance | 0.000717 | 0.00720 | 0.000772 | 0.00121 | 0.00318** | -0.000657 | 0.00952 |
|  | (0.000589) | (0.00595) | (0.000883) | (0.00139) | (0.000873) | (0.00165) | (0.00925) |
| TLU | 0.00132 | 0.0128 | -0.00739 | -0.00474 | -0.00714 | 0.00934** | 0.131** |
|  | (0.00463) | (0.0457) | (0.00491) | (0.00386) | (0.00597) | (0.00294) | (0.0482) |
| Irrigation at baseline | 0.00974 | 0.107 | -0.00101 | 0.0135 | -0.0472 | -0.0856 | 0.982*** |
|  | (0.0237) | (0.236) | (0.0588) | (0.0406) | (0.0429) | (0.0736) | (0.205) |
| Water source | -0.00398 | -0.0421 | -0.0454 | 0.0403 | 0.0381 | 0.139*** | 0.0331 |
|  | (0.0240) | (0.241) | (0.0745) | (0.104) | (0.0810) | (0.0328) | (0.248) |
| Climate shock | -0.0135 | -0.124 | 0.0294 | -0.0177 | 0.00729 | -0.0262 | -0.115 |
|  | (0.0361) | (0.352) | (0.0488) | (0.0650) | (0.0659) | (0.0274) | (0.177) |
| Idiosyncratic shock | 0.0486*** | 0.482*** | -0.0725 | -0.0329 | -0.0567 | -0.0430 | 0.715* |
|  | (0.0113) | (0.110) | (0.0405) | (0.0641) | (0.0486) | (0.0462) | (0.281) |
| Constant | 0.733*** | 7.324*** |  |  |  |  | 5.929*** |
|  | (0.0140) | (0.159) |  |  |  |  | (0.557) |
| Observations | 378 | 378 | 378 | 378 | 378 | 378 | 378 |
| R-squared | 0.112 | 0.113 |  |  |  |  | 0.307 |
| Robust standard errors in parentheses | | |  |  |  |  |  |
| *** p<0.01, ** p<0.05, * p<0.1 | |  |  |  |  |  |  |
| Note: Village dummies included, marginal effects reported for logistic regressions | | | | | |  |  |

1. Data to calculate two sub-indicators—autonomy in income and respect in the household—were not collected. [↑](#footnote-ref-1)
